# Alkalinity-Dependent Dual Role of Sodium Sulfate in Alkali-Activated Slag: From Synergistic Activation to Competitive Inhibition

**DOI:** 10.3390/ma19102177

**Published:** 2026-05-21

**Authors:** Nan Ding, Zhenyun Cheng, Jinghan Wu, Hua Lei, Meng Su, Bo Fu

**Affiliations:** 1 College of Civil Engineering, North Minzu University, Yinchuan 720021, China; 20247585@stu.nmu.edu.cn (N.D.);; 2 College of Civil Engineering, Hefei University of Technology, Hefei 230009, China

**Keywords:** alkali-activated slag cement, alkali-activated materials, slag activation, hydration kinetics, sodium sulfate, alkali equivalent, ettringite, monosulfate, ionic strength, C-(A)-S-H

## Abstract

Sodium sulfate-activated slag cement is considered a highly promising low-carbon cementitious material; however, its application is limited by low early-age activation efficiency and slow strength development. This study aims to systematically elucidate the coupled regulatory mechanism of alkalinity (2% and 4% Na_2_O equivalent) and sodium sulfate dosage on the performance of alkali-activated slag (AAS). Under standard curing conditions (20 ± 2 °C, relative humidity ≥ 95%), the macroscopic properties of the samples (workability, setting time, and compressive strength) and the evolution of their microstructure (analyzed by XRD, FTIR, and SEM-EDS) were evaluated. The results indicate that the effect of sodium sulfate on alkali-activated slag (AAS) strongly depends on the alkalinity. Under low-alkalinity conditions (2% Na_2_O), sodium sulfate exhibits a synergistic activation effect by increasing the ionic concentration, promoting slag depolymerization and the nucleation of ettringite (AFt). Specifically, compared with the control, incorporating 6 wt% sodium sulfate (N2S6 mix) increased compressive strength by approximately 82% at 3 days and 21% at 28 days. In contrast, under high-alkalinity conditions (4% Na_2_O), excessive sodium sulfate (≥2 wt%) shows an inhibitory effect. This is likely because an excess of sodium sulfate interferes with the normal polymerization pathways of the aluminosilicate network, suppressing the formation of the primary C-(A)-S-H gel and thus significantly reducing later-age strength. Microstructural analysis revealed that the hydration products in the composite-activated system mainly consist of C-(A)-S-H gel, ettringite (AFt), monosulfate (AFm), and hydrotalcite. This study investigates the observed kinetic trends of anion-competitive hydration under different alkalinity conditions, providing a theoretical basis for the mix design of low-carbon alkali-activated materials and the valorization of coal chemical industrial salts.

## 1. Introduction

The traditional cement industry faces severe challenges in achieving sustainable development due to its substantial carbon footprint and high resource consumption [1]. Consequently, many novel green cementitious materials have been developed as alternatives to Portland cement. Such as alkali-activated cement (AAC) (note: standard chemical symbols are used for dissolved ions and raw materials, such as Ca^2+^, Al^3+^, and Na_2_SO_4_; for complex hydration products, cement chemistry abbreviations are retained, e.g., C-(A)-S-H represents calcium aluminosilicate hydrate, and AFt represents ettringite) [2], magnesium silicate hydrate cement (M-S-H) [3], and limestone calcined clay cement (LC3) [4]; among these, AAC is considered the most promising alternative. AAC is typically composed of precursors and alkaline activators [5]. Ground granulated blast furnace slag (GBFS) is the most commonly used precursor [6], while commonly used alkalis include NaOH or sodium silicate [7]. Alkaline activators account for more than 70% of the carbon emissions of this type of cementitious material [8]. Sodium sulfate (Na_2_SO_4_) has evolved from a traditional “auxiliary activator” to a core regulatory tool in alkali-activated slag (AAS) design [9,10,11,12,13,14,15,16]. While traditional strong alkalis (NaOH, sodium silicate) offer high reactivity, they face challenges in safety and carbon footprint [17,18]. Recent studies have shifted towards low-alkalinity or near-neutral systems. For instance, Zheng et al. [17] systematically compared various composite combinations, marking a transition from empirical recipe optimization to rational systematic design. Yue et al. [19] further demonstrated that the synergy between sulfates and C-(A)-S-H gel is the key to balancing early-age kinetics and long-term durability. To overcome these limitations, researchers have proposed various composite activation strategies to achieve performance optimization. Al-Kroom et al. [20] found that nano magnesium oxide can accelerate the early hydration rate of sodium sulfate-activated slag and improve its early compressive strength. Dai et al. [21] found that using sodium sulfate alone as an activator leads to prolonged setting time and insufficient early compressive strength of slag mixtures, whereas the incorporation of sodium hydroxide can significantly improve the rheological properties, setting behavior, reaction kinetics, and mechanical performance of the slag system. Akturk et al. [22] investigated sodium carbonate as the primary activator for ground granulated blast furnace slag and found that its slow activation mechanism can be optimized by adding sodium hydroxide and a small amount of calcium hydroxide. The results showed that the incorporation of sodium hydroxide and calcium hydroxide enhances the dissolution rate of slag, thereby accelerating strength development. Wang et al. [23] investigated the effect of a combination of calcium oxide and sodium carbonate on the hydration reactions of alkali-activated slag cementitious materials. The results showed that the combination of CaO and Na_2_CO_3_ significantly increased the amount of hydration products, leading to a denser microstructure and higher compressive strength. Dai et al. [24] found that calcium hydroxide can significantly increase the early reaction rate of sodium sulfate-activated ground granulated blast furnace slag, shorten the setting time, and enhance early compressive strength. However, at high Ca(OH)_2_ contents (>1%), a more porous microstructure may be observed at later hydration stages. Previous studies have shown that sodium sulfate composite activators can effectively activate slag reactivity and improve material performance. It is worth noting that the application of sodium sulfate as a component of composite activators in slag-based cementitious materials aligns with the modern construction industry’s demand for sustainable development. The use of sodium sulfate as a substitute for highly alkaline activators not only reduces dependence on the energy-intensive and high-carbon-emission cement industry but also provides an effective pathway for the valorization of industrial waste [25,26]. This approach not only improves the overall performance of the material but also effectively reduces production costs and environmental burdens, demonstrating significant ecological benefits and promising engineering application prospects [27].

Previous studies have shown that sodium sulfate composite activators can effectively enhance slag reactivity. However, current research often focuses on single-variable optimization—either fixing the alkalinity to evaluate the sulfate dosage or vice versa. This fragmented approach has led to contradictory reports in the literature regarding the efficacy of sodium sulfate: some studies report significant early strength improvements, while others observe severe hydration retardation and macroscopic strength deterioration. A critical analysis of these discrepancies reveals a clear scientific gap: there is currently a lack of coupled quantification of alkalinity–sulfate dosage thresholds. Although the fundamental interaction between sulfate activators and alkalinity in alkali-activated slag (AAS) systems has been conceptually discussed in the literature, the boundary conditions at which sulfates transition from synergistic activators to competitive inhibitors remain to be fully explored. Therefore, this study evaluates potential threshold ranges through parametric analysis, advancing the understanding of AAS systems. By evaluating the coupled effects of different alkalinity levels (2% and 4% Na_2_O equivalent) and sodium sulfate dosages, this work identifies the relevant dosage ranges governing competitive hydration pathways. The scientific contribution of this study lies in characterizing the kinetic evolution of anion-competition-driven hydration and identifying experimental threshold behaviors where synergistic promotion shifts toward competitive inhibition, thereby helping to reconcile discrepancies in the existing literature. Such parameterization provides a useful theoretical framework for the mix design of low-carbon alkali-activated materials and supports the safe and large-scale utilization of industrial by-products.

## 2. Experiment

### 2.1. Raw Materials

**Figure 1:**
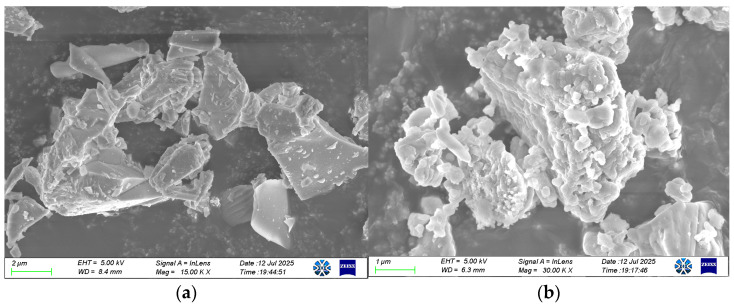
SEM micrographs of raw materials. (**a**) Images of micromorphology of GBFS [28]. (**b**) Images of micromorphology of Na_2_SO_4._

**Figure 2:**
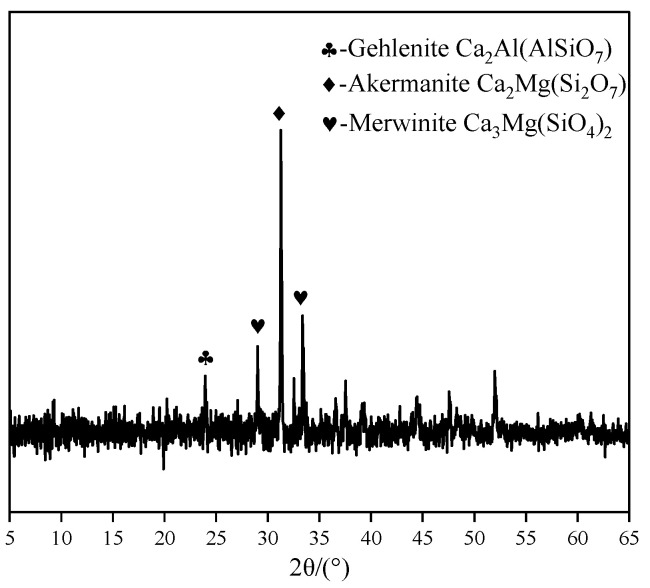
X-ray diffraction pattern of GBFS [28].

Ground granulated blast furnace slag (GBFS, S95), a light grayish-white powder, was obtained from Ningxia Qingtongxia Cement Co., Ltd. (Qingtongxia, China). Sodium hydroxide (analytical grade) was used as the primary activator and was purchased from Ningxia Tianshengyuan Biotechnology Co., Ltd. (Shizuishan, China). Sodium sulfate (analytical grade) was purchased from Tianjin Dingshengxin Chemical Co., Ltd. (Tianjin, China). As shown in Figure 1, the microscopic morphologies of the raw materials are presented. The GBFS particles exhibit an amorphous state. Na_2_SO_4_ crystals mainly appear as irregular block-shaped and short columnar aggregates, consisting of multilayered, plate-like crystals stacked together, with obvious layered step-like structural features on the crystal surfaces. The XRD pattern of GBFS is shown in Figure 2. As can be seen, a broad hump in the range of 25° and 35° (2θ) is clearly observed, indicating the presence of a glassy (amorphous) phase, which suggests that GBFS possesses potential reactivity.

**Figure 3:**
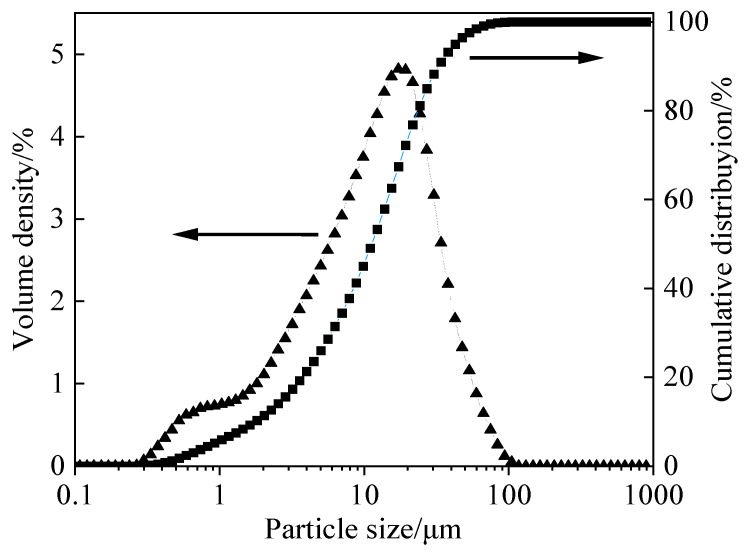
Particle size distribution of GBFS [28].

The chemical composition of the raw materials was characterized using an X-ray fluorescence (XRF, Zetium, Malvern Panalytical, Malvern, UK). The XRF results (Table 1) show that GBFS is mainly composed of SiO_2_, CaO, Al_2_O_3_, and MgO, which together account for 94.64% of the total mass fraction. The particle size distribution of GBFS was measured using an intelligent laser particle size analyzer (Bettersize 2000, Bettersize Instruments Ltd., Changsha, China). As shown in Figure 3, the GBFS particles exhibit a continuous size distribution, with a median particle size (D50) of 12.64 μm and a specific surface area of 450 m^2^/kg.

### 2.2. Sample Preparation

#### 2.2.1. Mix Proportions

The mix proportions of AAS are shown in Table 2. GBFS was used as the solid precursor, while sodium hydroxide (NaOH) and sodium sulfate (Na_2_SO_4_) were used as activators. The water-to-binder ratio (*w*/*b*) for all mixtures was 0.38. An alkaline solution was prepared using NaOH pellets and deionized water. The alkali equivalent levels were set at 2% and 4% Na_2_O (by mass of GBFS), corresponding to NaOH solution concentrations of approximately 1.3 mol/L and 2.6 mol/L (based on solution volume). Based on preliminary optimization and existing literature, 4% Na_2_O represents the standard activation level required to ensure early structural strength, while 2% Na_2_O serves as a low-alkalinity, low-carbon reference baseline for systematically evaluating the compensatory activation mechanism of sodium sulfate [29,30]. Prior to paste preparation, pre-weighed NaOH pellets were added to deionized water and continuously stirred until fully dissolved. The resulting solution was then sealed with plastic wrap to minimize carbonation and cooled to room temperature (20 ± 2 °C). On this basis, Na_2_SO_4_ was added at 0%, 1%, 2%, 4%, 6%, 8%, and 10% by mass of GBFS and fully dissolved under magnetic stirring to form a homogeneous NaOH–Na_2_SO_4_ composite activator solution. Although conventional sulfate dosages rarely exceed 6%, exploring higher dosages (up to 10%) is crucial for simulating the large-scale utilization of high-concentration industrial by-products, particularly mixed salts from coal chemical processes. This wide range helps to determine the absolute tolerance limit at which sulfate transitions from an activator to an inhibitor, as well as the precise kinetic thresholds. The total concentrations of Na^+^ and SO_4_^2−^ in each mix proportion system are listed in Table 2.

#### 2.2.2. Preparation of Mortar Specimens

Mortar specimens were prepared according to the mix proportions shown in Table 2. The binder-to-sand ratio was 1:2, and standard sand (ISO 679 [31]) was used. The specimens were mixed using a planetary mixer. The mixing procedure followed the Chinese national standard GB/T 17671-2021 [32], with the only modification being that the mixing water specified in the standard was replaced by the Na_2_SO_4_–NaOH composite activator solution. After mixing, the fresh mortar was cast into 40 mm × 40 mm × 40 mm molds, compacted on a vibrating table, and covered with plastic film to reduce moisture evaporation. The specimens were demolded after 24 h of curing in a standard curing room (20 ± 2 °C, relative humidity ≥ 95%) and then further cured under the same conditions until the designated testing ages.

#### 2.2.3. Preparation of Paste Specimens

The mix proportions of AAS are shown in Table 2. Ground granulated blast furnace slag (GBFS) was used as the solid precursor, while sodium hydroxide (NaOH) and sodium sulfate (Na_2_SO_4_) served as activators. The water-to-binder ratio (*w*/*b*) for all specimens was fixed at 0.38. An alkaline solution was prepared using NaOH pellets and deionized water. The alkali equivalent levels were set at 2% and 4% Na_2_O (by mass of GBFS). Based on the solution volume, the corresponding NaOH concentrations were approximately 1.3 mol/L and 2.6 mol/L, respectively. Before paste preparation, the pre-weighed NaOH pellets were added to deionized water and stirred continuously until completely dissolved. The resulting NaOH solution was then sealed with plastic film to minimize carbonation and cooled to room temperature (20 °C). On this basis, Na_2_SO_4_ was added at dosages of 0%, 1%, 2%, 4%, 6%, 8%, and 10% by mass of GBFS and stirred under magnetic agitation until completely dissolved, forming a homogeneous NaOH–Na_2_SO_4_ composite activator solution. The total concentrations of Na^+^ and SO_4_^2−^ in each mix proportion system are listed in Table 2.

### 2.3. Test Methods

The flowability of fresh paste was measured in accordance with the Chinese national standard GB/T 8077-2012 [33]. The prepared paste was quickly poured into a truncated cone mold, and the surface was leveled using a spatula. The mold was then lifted vertically to allow the paste to flow freely on a smooth glass plate. After 30 s, the maximum spread diameters in two perpendicular directions were measured, and the average value was taken as the flowability. Each mix was tested three times, and the results were reported as mean ± standard deviation. The setting time was measured using a Vicat apparatus in accordance with GB/T 1346-2011 [34]. Two Vicat apparatuses were used simultaneously during the test. The initial and final setting times were taken as the average of two measurements. If the difference between the two results exceeded 15 min, the test was repeated. The compressive strength was tested on mortar specimens. Before testing, the specimens were removed from the standard curing room, and surface moisture was wiped off. An automatic compression testing machine was used to apply axial loading at a loading rate of 2400 ± 200 N/s until failure of the specimens. The maximum failure load was recorded and used to calculate the compressive strength. For each mix proportion, six specimens were prepared at the specified curing ages (1, 3, 7, and 28 days). Outliers were defined as individual test results deviating by more than ±10% from the average of the six specimens. After excluding outliers, the average of at least four valid results was taken as the final compressive strength, and all results were reported as mean ± standard deviation.

For microstructural analyses (XRD, FTIR, TG-DTG, and SEM), hardened paste specimens at the specified curing ages were broken into small pieces. To terminate hydration, the fragments were immediately immersed in anhydrous isopropanol for 48 h, followed by vacuum drying at 40 °C for 24 h. It should be noted that the peak deconvolution of FTIR spectra provides a semi-quantitative distribution of structural units, and the reliability of these inferences was cross-validated by the phase evolution observed in XRD and TG-DTG analyses.

X-ray diffraction (XRD) analysis was performed using a SmartLab SE X-ray diffractometer (Rigaku Corporation, Tokyo, Japan). The testing conditions were as follows: tube voltage of 40 kV, tube current of 200 mA, Cu Kα radiation, scanning rate of 1°/min, and a 2θ scanning range of 5–65°.

Fourier transform infrared spectroscopy (FTIR) analysis was conducted using a Nicolet iS50 FTIR spectrometer (Thermo Fisher Scientific, Waltham, MA, USA). The samples were prepared using the KBr pellet method, in which 1 mg of sample powder was thoroughly mixed with 100 mg of KBr and pressed into pellets. The scanning range was 4000–400 cm^−1^, with a spectral resolution of ≤0.09 cm^−1^ and 32 accumulated scans.

Thermogravimetric–differential thermogravimetric (TG-DTG) analysis was performed using an STA 449 F3 simultaneous thermal analyzer (Netzsch Instrument GmbH, Selb, Germany). The temperature range was 30–1000 °C, with a heating rate of 10 °C/min. During the test, argon was used as the purge gas (flow rate: 50 mL/min), and nitrogen was used as the protective gas (flow rate: 20 mL/min).

Scanning electron microscopy with energy-dispersive X-ray spectroscopy (SEM–EDS) analysis was performed using a SIGMA 500 field emission scanning electron microscope integrated system (Carl Zeiss, Oberkochen, Germany).

### 2.4. Statistical Analysis

To ensure the reliability of macroscopic test results (flowability, setting time, and compressive strength), all physical property measurements were performed in at least three independent replicates. Data are presented as mean ± standard deviation (SD). Statistical significance between different mix proportions was evaluated using one-way analysis of variance (One-way ANOVA) followed by Tukey’s post hoc test. A *p*-value < 0.05 was considered statistically significant.

## 3. Results and Discussion

### 3.1. Fluidity

**Figure 4:**
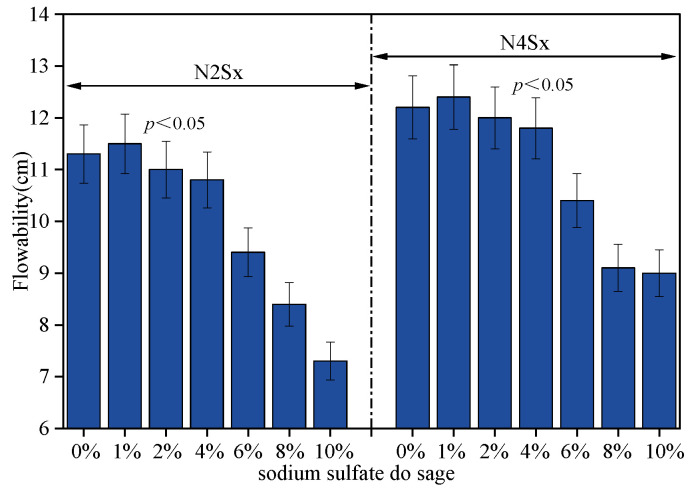
Effects of alkali equivalent (Na_2_O%) and Na_2_SO_4_ dosage on the fluidity of AAS.

Figure 4 shows the variation in flowability of Na_2_SO_4_–NaOH–slag composite pastes with Na_2_SO_4_ dosage under different alkali equivalent conditions. As shown, under alkali equivalent levels of 2% and 4% Na_2_O, the incorporation of 1% Na_2_SO_4_ effectively improves the flowability of the system, showing a statistically significant increase compared with the control group (one-way ANOVA, *p* < 0.05). However, when the Na_2_SO_4_ dosage exceeds 1%, the flowability exhibits a statistically significant decreasing trend with increasing sodium sulfate content (*p* < 0.05). These significant macroscopic changes are intrinsically related to the early-age water dynamics and the competitive formation of initial hydration products, which will be discussed in detail in Section 4.

### 3.2. Setting Time

**Figure 5:**
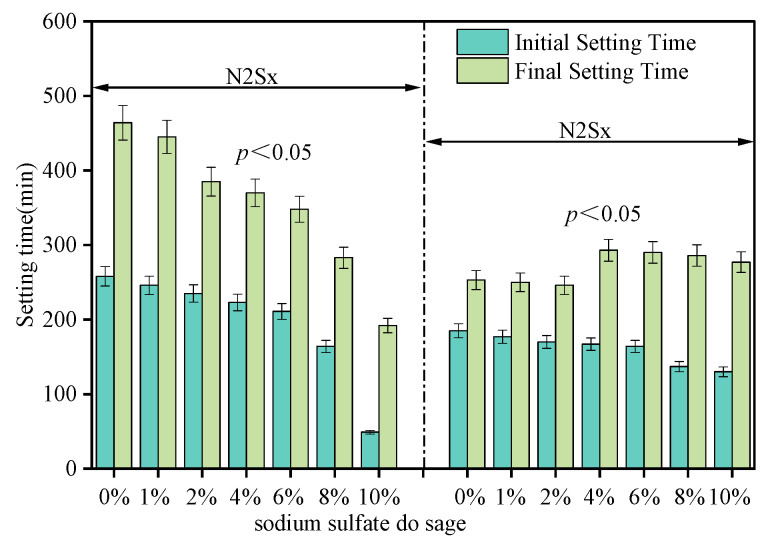
Effects of alkali equivalent (Na_2_O%) and Na_2_SO_4_ dosage on the setting time of AAS.

Figure 5 shows the effect of alkali equivalent (Na_2_O%) and Na_2_SO_4_ dosage on the setting times of alkali-activated slag. Under low-alkali conditions (2% Na_2_O), the incorporation of Na_2_SO_4_ significantly accelerates the hydration process (*p* < 0.05); as the dosage increases from 0% to 10%, the initial setting time decreases from 258 ± 12 min to 49 ± 5 min, while the final setting time shortens from 464 ± 15 min to 192 ± 8 min. Under high-alkali conditions (4% Na_2_O), when the Na_2_SO_4_ dosage increases from 0% to 2%, the initial setting time decreases from 185 min to 170 min, and the final setting time shortens from 253 min to 246 min.

When the Na_2_SO_4_ dosage further increases to 4%, the final setting time is significantly prolonged, rising from 246 min to 293 min. Subsequently, with a further increase in dosage, the final setting time gradually shortens. However, the initial setting time shows a continuous decreasing trend over the entire dosage range, dropping from 185 min to 130 min. The macroscopic variations in setting times—from initial acceleration to a complex, dosage-dependent retardation—indicate substantial changes in ionic strength and pore structure morphology. To avoid fragmented analysis, the potential acceleration and hydration-retarding mechanisms will be discussed in Section 4 in conjunction with microstructural data.

### 3.3. Compressive Strength

**Figure 6:**
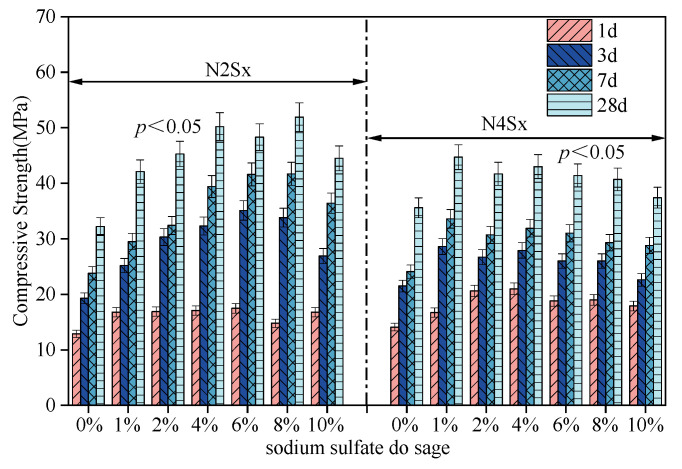
Effects of alkali equivalent and Na_2_SO_4_ dosage on the compressive strength of AAS mortar.

Figure 6 shows the variation in compressive strength of alkali-activated slag mortar under different alkali equivalent (Na_2_O%) levels and Na_2_SO_4_ dosages. Under low-alkali equivalent conditions (2% Na_2_O), as the Na_2_SO_4_ dosage increases, the compressive strength of the specimens at all curing ages shows a trend of initially increasing and then decreasing. At early ages, the incorporation of Na_2_SO_4_ is associated with enhanced strength development. The 1-day compressive strength shows a statistically significant increase (*p* < 0.05), rising from 12.9 ± 0.8 MPa in the control group (N2) to a peak value of 17.5 ± 1.2 MPa at a dosage of 6% (N2S6). Similarly, the 3-day and 7-day strengths also reach their maximum values at a 6% dosage, with increases of approximately 82% and 75%, respectively, compared with the control group (*p* < 0.05). At 28 days, the compressive strength reaches a maximum value of 50.2 MPa at a Na_2_SO_4_ dosage of 4% (N2S4). With further increases in dosage, the strength shows a decreasing trend, although it remains higher than that of the control group without Na_2_SO_4_. These results suggest that Na_2_SO_4_ may contribute to early-age strength development in low-alkali systems. Based on the overall performance, a suggested dosage range can be considered within approximately 4–6 wt%, where relatively favorable early-age and 28-day strengths are achieved while maintaining acceptable setting time and flowability.

At 28 days, the compressive strength reaches a maximum value of 50.2 MPa at a Na_2_SO_4_ dosage of 4% (N2S4). With further increases in dosage, the strength shows a decreasing trend, although it remains higher than that of the control group without Na_2_SO_4_. These results suggest that Na_2_SO_4_ may contribute to early-age strength development in low-alkali systems. Based on the overall performance, a suggested dosage range can be considered within approximately 4–6 wt%, where relatively favorable early-age and 28-day strengths are achieved while maintaining acceptable setting time and flowability. At 3, 7, and 28 days, the maximum compressive strengths are observed at a Na_2_SO_4_ dosage of 1%, reaching 28.6 MPa, 33.6 MPa, and 44.7 MPa, respectively. With further increases in Na_2_SO_4_ content, the medium- and long-term strength gradually decreases. These results indicate that, under high-alkali activation conditions, higher Na_2_SO_4_ dosages may be associated with short-term strength enhancement but less favorable strength development at later ages.

### 3.4. Phase Composition

#### 3.4.1. XRD

**Figure 7:**
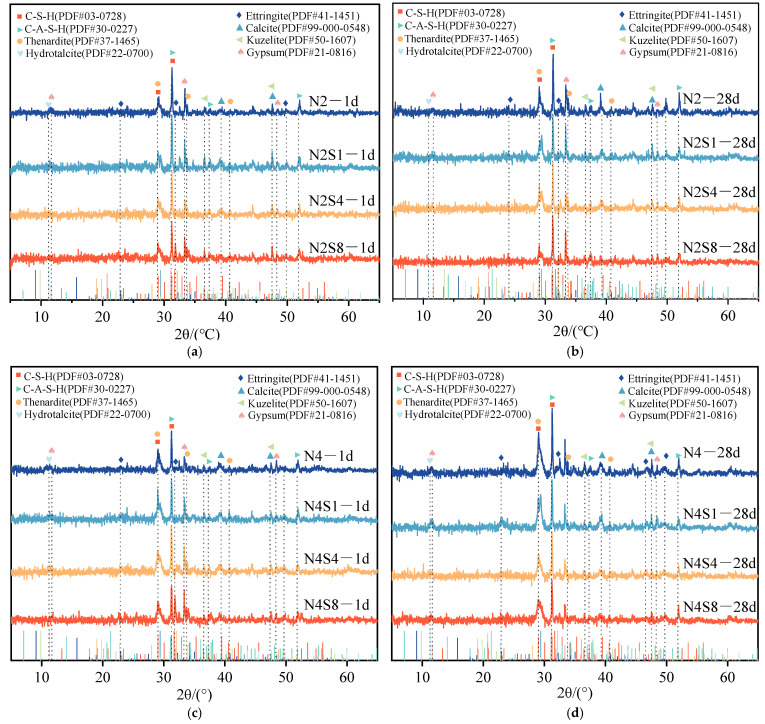
XRD patterns of AAS at 1 d and 28 d with varying alkali equivalents and Na_2_SO_4_ dosages. (**a**) N2Sx—1 d; (**b**) N2Sx—28 d; (**c**) N4Sx—1 d; (**d**) N4Sx—28 d [28].

Figure 7 shows the XRD patterns of the hardened pastes at 1-day and 28-day curing ages. The XRD patterns indicate that the hydration products of the specimens are mainly composed of C-(A)-S-H gel (PDF#03-0728), hydrotalcite (PDF#22-0700), ettringite (AFt) (PDF#41-1451), monosulfate-type calcium aluminate sulfate (Kuzelite/AFm) (PDF#50-1607), and calcite (PDF#99-000-0548). The presence of hydrotalcite diffraction peaks indicates that Mg^2+^ and Al^3+^ participate in the formation of layered double hydroxides (LDHs) [35]. The appearance of calcite may be attributed to impurities in the raw materials or carbonation processes [36]. It is noteworthy that no distinct AFt characteristic diffraction peaks are observed in some samples. This may be due to ettringite existing in the form of nanocrystals or low-crystallinity phases, whose signals are masked by the broad amorphous peak of the C-(A)-S-H gel. However, complementary evidence for the presence of these phases was provided by the mass loss observed in TG–DTG within the 50–100 °C range and by the needle-like morphologies identified in SEM analysis [37]. In a highly alkaline environment, the initially formed AFt is prone to transform into a more stable AFm phase [38]. The detection of Kuzelite diffraction peaks confirms that the sulfate–aluminate reaction pathway tends to favor the formation of the AFm phase [17]. In the Na_2_SO_4_-doped specimens, unreacted phases such as thenardite (PDF#37-1465) and gypsum (PDF#21-0816) were detected, indicating that the sulfate was not completely consumed.

In the low-alkali-equivalent (2% Na_2_O) system, the synergistic effect of Na^+^ and SO_4_^2−^ promotes slag dissolution, providing Ca, Si, and Al sources for the formation of C-(A)-S-H gel. XRD analysis indicates that, although the characteristic peaks of AFt are not prominent due to its low crystallinity, crystalline phases such as AFm and hydrotalcite are clearly detected and coexist with the primary gel matrix. The detailed mechanisms by which these phases contribute to the optimization of the microstructural framework through synergistic filling effects are discussed in Section 4.

In the high-alkali equivalent (4% Na_2_O) system, the strongly alkaline environment further promotes slag hydration, as evidenced by a higher intensity of the C-(A)-S-H diffraction peak compared with the low-alkali system. A small amount of Na_2_SO_4_ incorporation (N4S1) optimizes the microstructure by forming limited AFt and AFm crystals that fill gel pores [39]. Under highly alkaline conditions, excessive Na_2_SO_4_ leads to the substantial formation of AFm phases, and the associated volumetric changes during their formation may induce micro-defects [40]. Meanwhile, excessive SO_4_^2−^ ions may interfere with the optimal formation pathway of high-polymerization, low Ca/Si ratio C-(A)-S-H gel [41].

Comparison of the XRD patterns at 1 day and 28 days shows that with increasing curing age, the intensity of the C-(A)-S-H gel diffraction peaks significantly increases. This continuous accumulation of hydration products and phase development provides a direct mineralogical basis for the macroscopic strength gain of the hardened paste [42].

#### 3.4.2. FTIR

**Figure 8:**
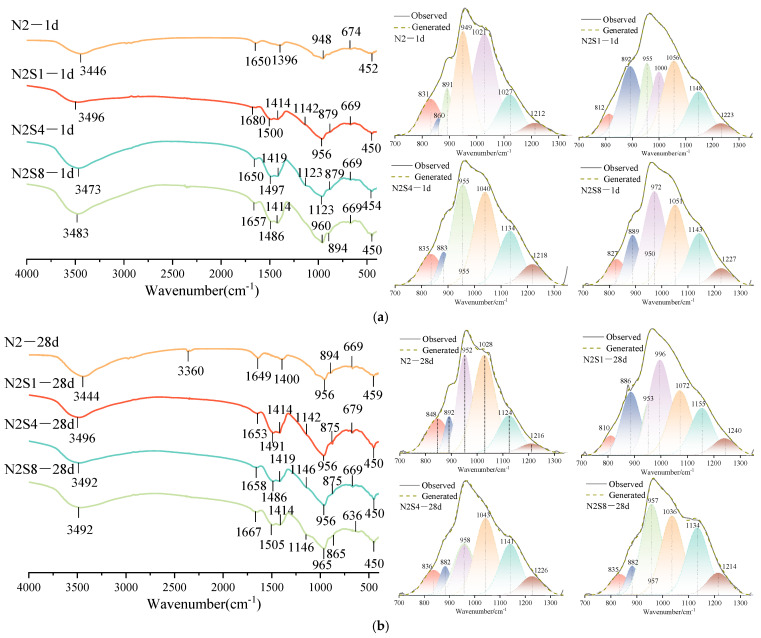

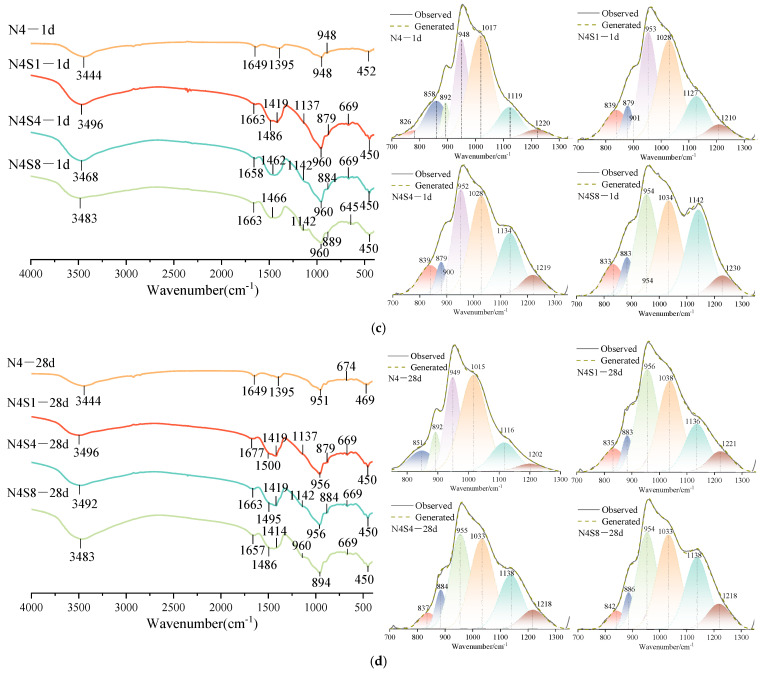
FTIR spectra of AAS at 1 d and 28 d with different alkali equivalents and Na_2_SO_4_ dosages. (**a**) N2Sx—1 d; (**b**) N2Sx—28 d; (**c**) N4Sx—1 d; (**d**) N4Sx—28 d.

Figure 8 presents the FTIR spectra of the hardened pastes under different alkali equivalent levels and Na_2_SO_4_ dosages at 1 day and 28 days of curing. Based on the spectral analysis, the main absorption bands can be assigned as follows: the peaks at around 1650 cm^−1^ and 3446 cm^−1^ correspond to the bending vibration of bound water molecules and the O-H stretching vibration in the precursor slag, respectively [43]. All samples exhibit distinct absorption bands in the ranges of 1600–1700 cm^−1^ and 3000–3500 cm^−1^, confirming the presence of chemically bound water associated with –OH groups in the system. The absorption peak at around 1414 cm^−1^ corresponds to the stretching vibration of the O-C-O bond in CO_3_^2−^ groups, indicating a certain degree of natural carbonation in the system [35]. The peak at 948 cm^−1^ is attributed to the asymmetric stretching vibration of Si-O-T (T = Si or Al) bonds [43].

The absorption bands observed in the 450–1100 cm^−1^ region are associated with the aluminosilicate network structures of N-A-S-H and C-(A)-S-H gels. The main absorption peak (around 948 cm^−1^) shifts toward higher wavenumbers, which generally indicates an increase in the degree of cross-linking of the silicate network and the formation of low Ca/Si ratio silicate phases [43]. The experimental results show that, compared with the control group, the samples containing Na_2_SO_4_ exhibit a significantly enhanced absorption peak intensity in this region, along with a shift of the wavenumber toward lower frequencies. This indicates that the incorporation of Na_2_SO_4_ promotes the formation of denser N-A-S-H and C-(A)-S-H gels [44], Specifically, the main Si-O-T absorption band shifts from approximately 948 cm^−1^ in the reference sample to 942 cm^−1^ at the optimal dosage, reflecting changes in the connectivity of the silicate network and an enhancement of the microstructural compactness. This conclusion is consistent with the XRD results and represents a key microstructural mechanism underlying the improvement in material strength.

In the samples with Na_2_SO_4_ incorporation, a new absorption peak at approximately 1137 cm^−1^, attributed to the stretching vibration of S–O bonds, was observed [45,46]. This peak is mainly associated with the formation of AFt [47] and may also include contributions from AFm phases. In the low-wavenumber region of 614–674 cm^−1^ and 445–516 cm^−1^, all samples exhibited absorption bands attributed to Mg-O and Al-O bond vibrations, indicating the formation of hydrotalcite-like phases in the system [48].

To further elucidate the effect of NaOH incorporation on the evolution of the silicate network structure, peak deconvolution was performed in the key spectral region of 800–1200 cm^−1^, and the fitting results are shown in Figure 8. This approach aims to resolve overlapping absorption bands and thus semi-quantitatively analyze the distribution of Q^n^ structural units with different degrees of polymerization, providing direct structural parameters for understanding the regulatory mechanism of Na_2_SO_4_.

**Figure 9:**
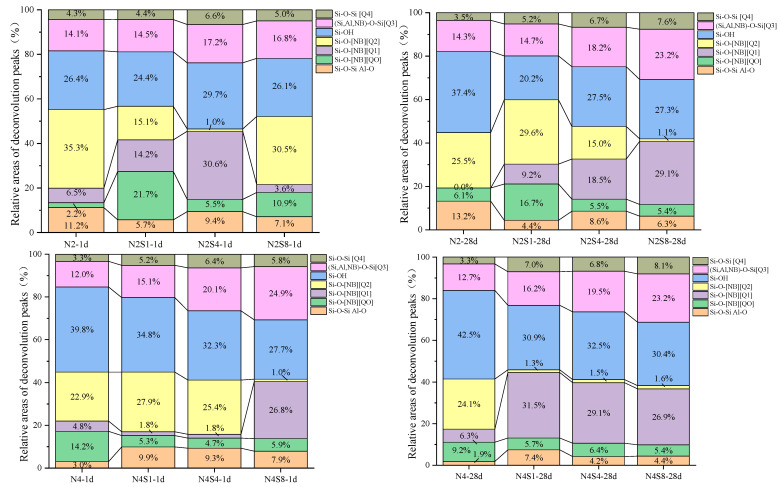
Relative distribution of Qn units from deconvoluted FTIR spectra of AAS at 1 d and 28 d.

Figure 9 presents the peak deconvolution results of the main Si-O-T (T = Si or Al) stretching vibration band (800–1300 cm^−1^) in the hardened pastes under different alkali equivalent levels and Na_2_SO_4_ contents. Gaussian curve fitting exhibited high reliability for all samples (R^2^ > 0.98), with the maximum relative fitting uncertainty of individual phase fractions being ±2.5%, enabling a quantitative characterization of the distribution of silicate Q^n^ structural units. The assignment of each deconvoluted peak was determined based on the existing literature.

The peak at 795–814 cm^−1^ is attributed to the symmetric stretching vibration of Si-O-Si in quartz and the Al-O stretching vibration in mullite-like structures [49].

The peak at 850–880 cm^−1^ is assigned to Si-O^−^ (non-bridging oxygen) vibrations and Si-O vibrations of isolated silicate units [Q^0^] structures [50].

The peak at 900–930 cm^−1^ is associated with dimer [Q^1^] structures [50].

The peak at 950–990 cm^−1^ corresponds to Si-O stretching vibrations of chain-like [Q^2^] structures [50,51].

The peak at around 980 cm^−1^ is attributed to Si-OH stretching vibrations [51].

The broad and intense band at 1048–1100 cm^−1^ is assigned to the asymmetric stretching vibration of (Si, Al, NBO)-O-Si, which is characteristic of layered [Q^3^] structures [50];

1162–1200 cm^−1^ is attributed to the asymmetric stretching vibration of Si-O-Si in three-dimensional network Q^4^ structures [50].

In the low-alkali equivalent (2% Na_2_O) system, the incorporation of Na_2_SO_4_ significantly regulates the dissolution of slag and the reorganization of hydration products at early ages. At 1 day of curing, the addition of a small amount of Na_2_SO_4_ (N2S1) reduced the proportion of Q^2^ units from 35.3% in the reference sample (N2) to 15.1%, while the low-polymerization Q^0^ + Q^1^ units increased from 8.7% to 35.9%, and the Si-O-Si/Al-O bonds decreased from 11.2% to 5.7%. When the Na_2_SO_4_ dosage increased to 4% (N2S4), peak deconvolution results showed that the proportion of Q^2^ structural units decreased to 1.0%, while that of Q^1^ units increased to 30.6%, accompanied by an increase in Si-OH content. This spectral shift suggests that the system is predominantly characterized by intermediate chain-like structural units. The relationships between these structural distributions and the initial depolymerization kinetics of the aluminosilicate precursor, as well as the availability of polycondensation active sites, are comprehensively discussed in Section 4. When the dosage increased to 8% (N2S8), the Q^2^ fraction rose to 30.5%, while Q^1^ units significantly decreased. This may be attributed to the competitive adsorption of excessive SO_4_^2−^ ions, which inhibits the coordination rearrangement of Al and induces the precipitation of metastable aluminosilicate phases, thereby altering the evolution pathway of the silicate network structure [52]. This is consistent with the XRD results, indicating that different mineral phases may have formed in the system [53]. At 28 days of curing, with an appropriate dosage of sodium sulfate (N2S4), the proportions of Q^3^ (18.2%) and Q^2^ (15.0%) units were optimized, Q^4^ units slightly increased, and the Si-OH content decreased, while the Si-O-Si/Al-O bonds remained at a relatively high level. This reflects the formation of a more highly polymerized and denser network structure in the system [54]. In contrast, excessive sodium sulfate incorporation (N2S8) led to an increase in the Q^0^ + Q^1^ content and a lower Q^2^ fraction, indicating the formation of a more disordered network structure [52].

In the high-alkali equivalent (4% Na_2_O) system, the structural optimization effect of Na_2_SO_4_ on the silicate network exhibits a clear dosage-dependent regime. At 1 day of curing, the reference sample (N4) was mainly characterized by Q^2^ units (22.8%) and a relatively high Si-OH content (39.8%), while the Si-O-Si/Al-O bond content was low. This indicates that in the high-alkali system without sodium sulfate incorporation, the initial hydration products exhibit a relatively low degree of polymerization and a high content of silanol groups [55]. After the incorporation of 1% sodium sulfate (N4S1), the Q^2^ content increased to 27.9%, the Si-O-Si/Al-O bond content increased, and the Si-OH content decreased, indicating that a small amount of sodium sulfate promotes the initial polymerization of the silicate network and the formation of bridging oxygen bonds [56]. As the sodium sulfate dosage increased to 4% (N4S4), the contents of Q^3^ and Q^4^ units increased, indicating a further enhancement in the degree of polymerization of the system. When the dosage increased to 8% (N4S8), although the cross-linking degree remained relatively high, the Q^2^ fraction decreased significantly, and the Si-O-Si/Al-O bond content also declined. This suggests that excessive sulfate incorporation disrupts the stable formation of Si-O-Si/Al-O bridging bonds, resulting in a highly polymerized but structurally defective network [57]. At 28 days of curing, the incorporation of a small amount of sodium sulfate (N4S1) optimized the distribution of Q^2^ (31.5%) and Q^3^ (16.2%) units, significantly reduced the Si-OH content, and formed a dense network structure with good connectivity. In contrast, when the sodium sulfate dosage was excessive (N4S8), although the total content of highly polymerized units (Q^3^ + Q^4^) remained relatively high, the Q^2^ fraction was low and the Si-OH content remained elevated, while the Si-O-Si/Al-O bond content decreased. This indicates that the structural connectivity was compromised, potentially leading to a highly polymerized network with weak internal bonding [53].

#### 3.4.3. TG-DTG

**Figure 10:**
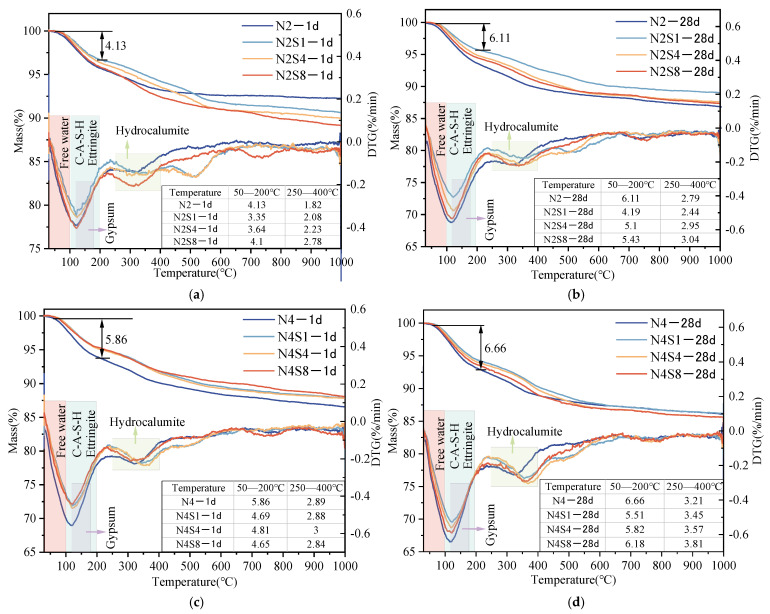
TG-DTG curves of AAS at 1 d and 28 d under different alkali equivalents and Na_2_SO_4_ dosages. (**a**) N2Sx—1 d; (**b**) N2Sx—28 d; (**c**) N4Sx—1 d; (**d**) N4Sx—28 d.

Thermogravimetric analysis (TG) was conducted on AAS pastes with different curing ages and Na_2_SO_4_ dosages to further elucidate the phase evolution, and the results are shown in Figure 10. DTG curves indicate that the main mass loss peak occurs between 50 °C and 150 °C. Although precise phase quantification is limited due to the overlapping thermal decomposition of C-(A)-S-H, AFt, and unreacted gypsum, a semi-quantitative estimation of this region shows that, depending on the alkalinity, the total mass loss ranges from approximately 4.13% to 6.66%, corresponding to the combined dehydration of these early hydration products [58]. The gradual mass loss observed in the 100–200 °C range is attributed to the continuous dehydration of C-(A)-S-H gel [58]. By comparing the DTG data at 1 day and 28 days, the total mass loss increases significantly with prolonged curing age, indicating a higher degree of hydration and accumulation of reaction products. The endothermic peak observed in the 250–400 °C range is generally associated with the decomposition of hydrotalcite [58]. The weak peaks appearing around 500 °C and 700 °C correspond to the decarbonation reactions of calcite (CaCO_3_) with different crystalline forms [58]. These thermogravimetric results are consistent with the phase analysis obtained from XRD.

Under a low-alkali equivalent (2% Na_2_O), the incorporation of Na_2_SO_4_ has a significant regulatory effect on the evolution of hydration products in AAS pastes. As shown in Figure 10a,b, at 1 day, the mass loss in the 50–200 °C range for the system without Na_2_SO_4_ (N2) is 4.13%, whereas it decreases to 3.35% after adding 1% Na_2_SO_4_ (N2S1). This phenomenon points to a shift in early phase assemblages. The specific competitive reactions between SO_4_^2−^, Al^3+^, and Ca^2+^ and how they physically interfere with the C-(A)-S-H gel network are integrated into the main discussion (Section 4) to correlate with macroscopic property changes. With increasing Na_2_SO_4_ dosage (e.g., N2S8), the formation of AFt and AFm compensates for part of the mass loss, leading to a partial recovery in the peak intensity within the 100–200 °C range; however, it still reflects significant fluctuations in phase composition.

Under a high-alkali equivalent (4% Na_2_O), the incorporation of Na_2_SO_4_ has a relatively limited effect on the overall mass loss behavior of the AAS system. As shown in Figure 10c,d, under highly alkaline conditions, a pronounced dehydration peak is observed in the 30–200 °C range regardless of whether Na_2_SO_4_ is added. The control group without Na_2_SO_4_ consistently exhibits the highest mass loss values (5.86% at 1 day and 6.66% at 28 days). This indicates that the high concentration of OH^−^ accelerates the dissolution of slag and the precipitation of C-(A)-S-H gel, leading to the dominance of the gel phase in the system [58]. Unlike the low-alkali equivalent condition, under highly alkaline conditions, although Na_2_SO_4_ can promote the formation of a small amount of AFt/AFm, its contribution to the total dehydration is much smaller than that of C-(A)-S-H gel. As a result, the DTG curves of the mixtures with different Na_2_SO_4_ dosages largely overlap, indicating that under a high activation level, the phase evolution of the system is predominantly governed by the alkali equivalent. This reduces the sensitivity of the system to variations in Na_2_SO_4_ content, and the incorporation of Na_2_SO_4_ exhibits only a weak auxiliary regulatory effect.

#### 3.4.4. SEM-EDS

**Figure 11:**
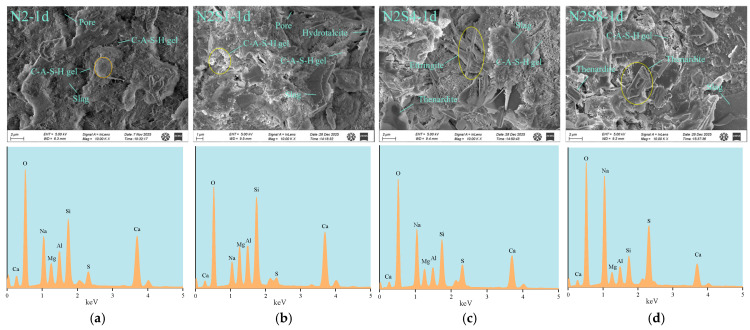

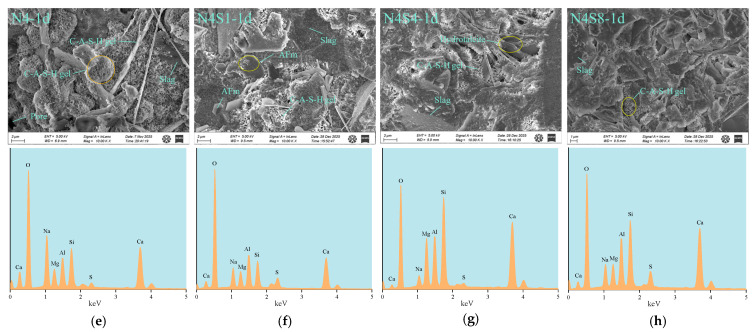
SEM-EDS Spectra of 1-day Hardened Paste under Different Alkali Equivalents-Sodium Sulfate Dosages (**a**) N2—1 d; (**b**) N2S1—1 d; (**c**) N2S4—1 d; (**d**) N2S8—1 d; (**e**) N4—1 d; (**f**) N4S1—1 d; (**g**) N4S4—1 d; (**h**) N4S8—1 d.

**Figure 12:**
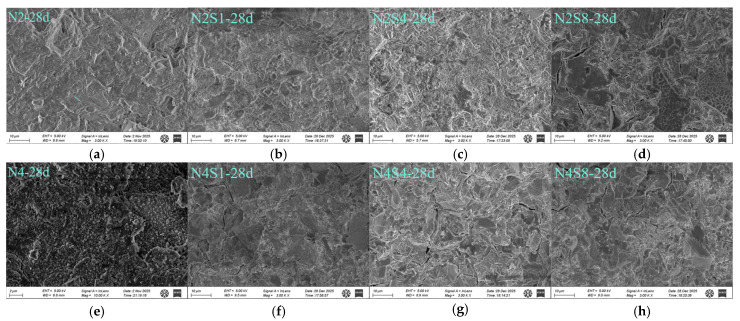
SEM images of 28-day hardened paste under different alkali equivalents and sodium sulfate dosages. (**a**) N2—28 d; (**b**) N2S1—28 d; (**c**) N2S4—28 d; (**d**) N2S8—28 d; (**e**) N4—28 d; (**f**) N4S1—28 d; (**g**) N4S4—28 d; (**h**) N4S8—28 d.

Figure 11 and Figure 12 present the SEM–EDS images of the slag–Na_2_SO_4_–NaOH system at 1 day and 28 days. Under a low-alkali equivalent (2%), as shown in Figure 11b,c, the incorporation of sodium sulfate promotes the formation of AFt/AFm at 1 day [39]. These needle- and rod-like crystals are interwoven with amorphous C-(A)-S-H gel, resulting in a visually denser microstructure. It should be noted that these SEM observations are qualitative; future studies combining image-based statistical analysis and Mercury Intrusion Porosimetry (MIP) are needed to obtain quantitative pore size distributions and statistically validate these microstructural refinements. As shown in Figure 11c,d, when the dosage of sodium sulfate is excessive, unreacted thenardite crystalline phases are observed, indicating that the excess Na_2_SO_4_ does not fully participate in the hydration reaction or become incorporated into the gel structure [59].

At 28 days, morphological observations indicate that the needle-like AFt and AFm crystals formed due to the incorporation of sodium sulfate act as micro-fillers, effectively occupying the gaps between micro-aggregates and contributing to a denser local microstructure [60]. As shown in Figure 12d, excessive incorporation of sodium sulfate may be associated with the development of microcracks, while the underlying mechanisms are discussed in Section 4.

Under a high-alkali equivalent (4%) condition, no obvious AFt or thenardite crystals are observed in the SEM images. However, as shown in Figure 11c, plate-like hydrotalcite minerals are found embedded within the C-(A)-S-H gel matrix. Compared with Figure 11a,e, the C-(A)-S-H gel formed under high-alkali equivalent conditions is denser than that under low-alkali equivalent conditions. This is because the high alkalinity promotes rapid dissolution of slag, leading to the accelerated formation of a silicoaluminate gel network at early ages [29]. This dense structure suppresses the long-term stability of crystalline phases such as AFt and may incorporate most sulfate ions into the gel structure through solid solution, thereby reducing sulfate crystallization and associated expansion [61]. Under high-alkali equivalent conditions, a dense gel framework is formed within the system, while the crystal-enhancing effect provided by sodium sulfate at an early age is relatively limited. As shown in Figure 12g,h, at 28 days, excessive sodium sulfate incorporation also induces microcracking; however, the extent of damage is comparatively lower. This may be attributed to the rapid dissolution of slag and the formation of a highly densified C-(A)-S-H gel network under highly alkaline conditions, which provides a stronger constraint and stress-dissipation capacity for internal stresses [62]. The dense structure can partially buffer these stresses; however, when the sodium sulfate dosage is excessive, the continuous crystallization pressure and potential expansive stress act synergistically, leading to microcrack propagation in locally weak regions.

## 4. Discussion

**Figure 13:**
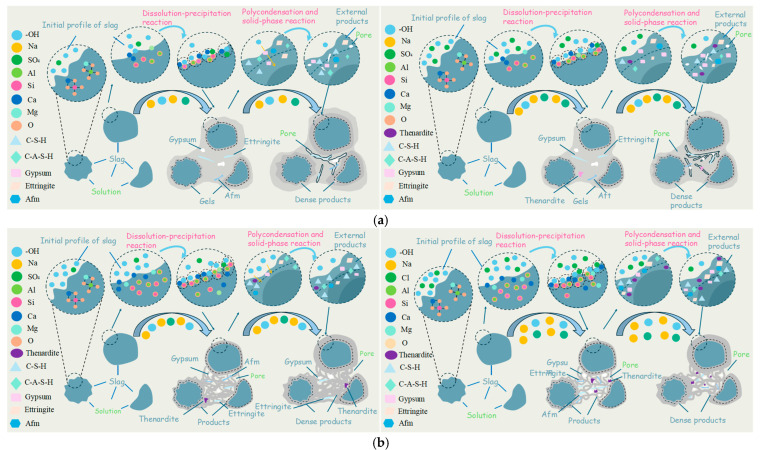
Schematic diagram illustrating the effects of alkali equivalent and Na_2_SO_4_ dosage on the slag reaction process. (**a**) Schematic of the hydration mechanism of low-alkali-equivalent AAS systems with different Na_2_SO_4_ dosages; (**b**) schematic of the hydration mechanism of high-alkali-equivalent AAS systems with different Na_2_SO_4_ dosages.

As shown in Figure 13, the synergistic effect may be associated with a complex, alkalinity-dependent competitive hydration pathway, which potentially links early-age water dynamics and phase assemblages with macroscopic performance. Under low-alkali equivalent conditions (2% Na_2_O), the incorporation of sodium sulfate primarily promotes slag dissolution by increasing the ionic strength of the pore solution. Although in situ measurements of early-age water dynamics were not directly conducted in this study, based on macroscopic test results and existing literature, the variation in the system’s flowability can be inferred to be closely related to the SO_4_^2−^ concentration-dependent formation of ettringite (AFt). At low Na_2_SO_4_ dosages, the specific competitive reactions observed in the early phase assemblages fundamentally dictate the system’s structural development; the limited SO_4_^2−^ competitively reacts with dissolved Ca^2+^ and Al^3+^ to promote the early formation of a small amount of AFt [1,63,64,65]. A generalized pathway for this early-stage precipitation from the aqueous pore solution can be represented as follows: 6Ca^2+^_(aq)_ + 2Al(OH)^4−^_(aq)_ + 3SO_4_^2−^_(aq)_ + 4OH^−^_(aq)_ + 26H_2_O_(l)_ → Ca_6_Al_2_(SO_4_)_3_(OH)_12_⋅26H_2_O_(s)_. It is speculated that this early competitive mechanism actively interferes with the optimal nucleation and growth of the C-(A)-S-H gel network, causing the initial mass loss fluctuations observed in the TG-DTG analysis. However, this slight delay in substantial C-(A)-S-H precipitation temporarily limits the increase in paste viscosity, manifesting as an improvement in macroscopic flowability. Simultaneously, the macroscopic setting time variations are driven by dynamic shifts in these early hydration kinetics. The increased ionic strength and AFt nucleation accelerate the overall slag dissolution and hydration process; as a result, the paste loses its plasticity, leading to a shortened setting time [66]. FTIR and XRD results indicate that an appropriate Na_2_SO_4_ dosage (≤6%) ultimately optimizes the C-(A)-S-H gel structure. This hypothesis is supported by cross-technique integration: the increased low-polymerization Q^1^/Q^2^ ratio observed in FTIR is directly associated with the initial structural breakdown phase, while the subsequent accumulation of hydration products (evidenced by a 4.13–6.66% mass loss in TG-DTG) and the qualitative morphological densification observed in SEM, coupled with the statistically significant mass loss (*p* < 0.05) identified in TG-DTG, provides a consistent mineralogical explanation for the significant (*p* < 0.05) early-age strength enhancement observed at the 4–6% dosage. In contrast, higher SO_4_^2−^ concentrations (>6%) promote the rapid formation of AFt [1,64], resulting in an interlocking network of needle-like crystals [40]. As reported in the literature, the development of this microstructure physically binds a large amount of free water and significantly increases the internal cohesion of the paste, ultimately leading to a reduction in macroscopic flowability [67]. Furthermore, this excessive crystallization and the presence of residual unreacted phases weaken the overall structural integrity, a trend further confirmed by SEM and TG-DTG analyses, causing strength degradation.

As shown in Figure 12d, excessive incorporation of sodium sulfate may also be associated with the propagation of microcracks. This behavior may be related to crystallization pressure and phase transformation of sodium sulfate within the pore structure, as well as expansive stresses generated by secondary reactions between free sulfate ions and early hydration products, which may contribute to the formation of expansive ettringite [59,68]. These microstructural changes may further compromise structural integrity and are consistent with the observed strength degradation.

Under high-alkali equivalent conditions (4% Na_2_O), the system is dominated by strong alkaline activation, leading to rapid formation of C-(A)-S-H gel that occupies the main reaction space. The macroscopic setting time variations under these conditions are equally complex. The incorporation of an appropriate amount of Na_2_SO_4_ can accelerate setting by promoting early hydration [21] and regulate the gel structure through the formation of limited AFt/AFm phases, thereby improving later-age performance. However, excessive addition (≥2%) may lead to excessively high ionic strength, which alters the morphology of hydration products and the pore structure, thereby hindering the transport of water and ions to the surface of unhydrated particles and slowing down the later hydration process [64]. FTIR deconvolution results suggest that when Na_2_SO_4_ ≥ 2%, it actively interferes with the evolution of the gel structure; the highly polymerized Q^3^/Q^2^ structural ratio tends to decrease, which appears to be associated with the observed reduction in 28-day compressive strength (from 44.7 MPa at 1% dosage to lower values). This indicates that excessive sulfate may hinder structural densification, potentially limiting the development of medium- to long-term strength. With further increases in Na_2_SO_4_ content (≥4%), the high concentration of SO_4_^2−^ dominates the hydration process through a coagulation-promoting effect, inducing rapid precipitation of hydration products and thus shortening the final setting time again [55,69]. In summary, the synergistic effect of sodium sulfate and sodium hydroxide strongly depends on the system’s alkalinity. Under low-alkalinity conditions, its role is primarily manifested through crystalline-phase formation and structural filling, whereas under high-alkalinity conditions, it mainly regulates the gel structure but with limited effectiveness. Excessive incorporation of Na_2_SO_4_ adversely affects structural stability in both systems, and the optimal dosage range is significantly constrained by the alkalinity level.

From both environmental and practical perspectives, the choice of activator in AAS systems has significant implications for CO_2_ emissions. Traditional high-alkalinity activators (e.g., concentrated NaOH or waterglass) contribute more than 70% of the carbon footprint of alkali-activated materials due to their energy-intensive production. The approach explored in this study—partially substituting strong alkalis with sodium sulfate, a widely available and low-cost industrial by-product—demonstrates a potential pathway to reduce embodied CO_2_ emissions while maintaining comparable early-age mechanical performance.

However, several limitations should be acknowledged. The present study primarily focuses on early-age to 28-day properties, and long-term durability—such as resistance to carbonation, freeze–thaw cycles, and external sulfate attack—was not evaluated. In addition, the mechanistic interpretations are based on conventional characterization techniques (XRD, FTIR, TG–DTG, and SEM–EDS), and the absence of advanced methods such as solid-state NMR or in situ pore solution analysis limits detailed structural resolution.

Therefore, future research should emphasize long-term performance evolution by extending curing durations and incorporating advanced characterization techniques to better resolve the structural features of C-(A)-S-H gel and sulfur-containing phases. The synergistic effects of Na_2_SO_4_ and NaOH on slag dissolution and hydration kinetics could be further clarified through combined approaches, such as in situ XRD, isothermal calorimetry, and pore solution chemistry analysis. In addition, the structural stability of the system under complex environmental conditions (e.g., temperature, humidity, carbonation, and sulfate exposure) should be systematically evaluated. Multiscale characterization integrated with thermodynamic and kinetic modeling is also needed to establish robust relationships between microstructure and macroscopic performance. Finally, from an engineering perspective, life-cycle assessment and large-scale validation are essential to optimize mix design and assess practical applicability.

These efforts will contribute to a more comprehensive understanding of composite activation systems and support the reliable, large-scale application of low-carbon alkali-activated materials.

## 5. Conclusions

This study aimed to investigate the coupled effects of different alkali equivalents and sodium sulfate dosages on the performance of AAS. The following key conclusions are drawn:

1. Sodium sulfate exhibits a pronounced dual role in AAS systems. Its effect on hydration reactions—whether promoting or inhibiting—appears to be largely influenced by the initial alkalinity of the system, rather than following a simple linear enhancement relationship.

2. Under low-alkalinity conditions (2% Na_2_O), sodium sulfate (≤6 wt%) acts as a synergistic activator by significantly increasing the ionic strength of the pore solution and promoting early-stage depolymerization of slag. The resulting AFt/AFm phases, together with the volumetric filling effect of C-(A)-S-H gel, lead to a substantial improvement in compressive strength at both 3 and 28 days.

3. Under high-alkalinity conditions (4% Na_2_O), the high concentration of OH^−^ dominates the early-stage rapid nucleation process. Sodium sulfate (≥2 wt%) acts as a competitive inhibitor, disrupting the normal polymerization pathway of the aluminosilicate network, suppressing the growth of the primary binding phase C-(A)-S-H, and consequently leading to reduced later-age densification and strength degradation.

4. In the Na_2_SO_4_–NaOH–slag composite system, the hydration products mainly include C-(A)-S-H gel, ettringite, AFm phases (such as kuzelite), and hydrotalcite. Under low-alkalinity conditions, an appropriate amount of sodium sulfate facilitates the synergistic filling of pores by crystalline phases and gels, leading to a denser and more homogeneous microstructure, which provides a mineralogical basis for improved macroscopic performance.

5. Regarding practical mix design recommendations, under the standard laboratory curing conditions used in this study, a sodium sulfate dosage of 4–6 wt% is recommended for low-alkalinity systems to achieve a synergistic improvement in both early-age and long-term strength. In high-alkalinity systems, the sodium sulfate content should be strictly limited to within 1 wt% to prevent performance deterioration due to anion competition effects. Future durability tests under realistic field conditions (e.g., carbonation, freezing-thawing, and external sulfate attack) are required to fully validate these findings for large-scale practical applications.

## Figures and Tables

**Table 1:** Chemical properties of GBFS [28].

Oxide	SiO_2_	MgO	Al_2_O_3_	Fe_2_O_3_	SO_3_	K_2_O	Na_2_O	CaO	Fe_2_O_3_	LOI
wt%	33.64	12.19	16.93	0.40	2.46	0.35	0.78	31.84	0.40	0.09

**Table 2:** Mixture design of alkali-activated slag mixtures.

No.	Na_2_O (wt%)	Na_2_SO_4_ (wt%)	[SO_4_^2−^] (mol/L)	[Na^+^] (mol/L)	GBFS	*w*/*b*
N2	2	-	0	0	1	0.38
N2S1		1	0.18	2.00		
N2S2		2	0.36	2.22		
N2S4		4	0.71	2.62		
N2S6		6	1.04	3.00		
N2S8		8	1.37	3.37		
N2S10		10	1.68	3.73		
N4	4	-	0	0		
N4S1		1	0.17	3.53		
N4S2		2	0.35	3.87		
N4S4		4	0.68	4.48		
N4S6		6	1.01	5.08		
N4S8		8	1.32	5.67		
N4S10		10	1.63	6.25		

Note: N2 and N4 represent the reference groups with alkali equivalents of 2% and 4% Na_2_O, respectively. S1, S2, S4, S6, S8, and S10 denote Na_2_SO_4_ dosages of 1%, 2%, 4%, 6%, 8%, and 10% by mass of slag, respectively.

## Data Availability

The original contributions presented in this study are included in the article. Further inquiries can be directed to the corresponding author.
